# Mammary Myofibroblastoma - An Elusive Cause of Breast Lump

**DOI:** 10.15190/d.2023.19

**Published:** 2023-12-31

**Authors:** Nilay Nishith, Sankalp Sancheti, Puneet Kaur Somal, Aishwarya Sharma, Ravikiran N Pawar

**Affiliations:** ^1^Department of Pathology, Homi Bhabha Cancer Hospital & Research Centre, New Chandigarh, Punjab, India

**Keywords:** Benign, breast, diagnosis, myofibroblastoma, neoplasm.

## Abstract

Mammary myofibroblastoma (MM) is an uncommon, benign mesenchymal neoplasm with a favourable prognosis. Its resemblance to various other benign and malignant lesions of the breast makes precise diagnosis challenging when examining biopsy samples. The rarity of mammary myofibroblastoma in India and worldwide underscores the importance of our case report, as we aim to contribute to the existing literature and expand the knowledge base of this neoplasm. Furthermore, we have delved into the diagnostic complexities associated with this lesion and highlighted the ancillary techniques employed to achieve an accurate and reliable diagnosis.

## Introduction

Mesenchymal tumours of the breast represent a rare and highly varied category of neoplasms, posing significant challenges for pathologists and clinicians alike. The initial diagnosis is complicated by the similarity in appearance between benign and malignant lesions, making it difficult to distinguish them. Mammary myofibroblastoma (MM) is one such uncommon benign mesenchymal tumour, often presenting difficulties in diagnosis due to its rarity and resemblance to various other breast lesions^1^. We present the case report of a 61-year-old female patient with a breast lump for 10 years. She was evaluated elsewhere and was misdiagnosed as a malignant neoplasm based on clinical and radiological findings. Conversely, her primary radiological assessment at our institute suggested a hamartomatous lesion. Following this she underwent wide local excision and a histopathological examination aided by immunohistochemistry led to a conclusive diagnosis of mammary myofibro-blastoma. In addition to the diagnostic intricacies, this case report also aims to highlight recent advances in understanding myofibroblastoma with emphasis on its molecular characteristics.

## Case report

A 61-year-old female presented with a long-standing lump in her right breast, persisting for a decade. Upon clinical examination, a palpable mass measuring 5 x 5 cm was identified in the upper inner quadrant of the right breast. The mass exhibited firm consistency, was non-tender, not adherent to the skin or deep structures, and showed no signs of nipple retraction or “peau d'orange” appearance. A lymph node in the right axilla, measuring approximately 2 x 1 cm, was also noted. The contralateral breast and axilla displayed no abnormalities. Initial haematological and biochemical tests yielded results within normal ranges. Subsequently, bilateral mammography unveiled a large, well-encapsulated lesion with heterogeneous, iso-to-hyperdense characteristics, measuring 8.4 x 6.6 cm in the upper central region of the right breast. Notably, there were no suspicious calcifications or architectural distortions detected. These findings were in favour of a hamartomatous lesion. Ultrasound of the ipsilateral axilla revealed an enlarged lymph node with eccentric cortical hypertrophy, measuring 2.3 x 1 cm, and displaying a cortical thickness of 6 mm. Following this, a biopsy of the right breast mass was conducted. The histopathological report was committed with descriptive findings, indicating the absence of granulomas, in-situ, or invasive malignancy. Furthermore, fine needle aspiration cytology of the ipsilateral lymph node was also performed, and the results indicated reactive lymphoid hyperplasia. Considering the clinical and radiological findings, as well as the negative biopsy report, the decision was made to proceed with a lumpectomy.

**Figure 1:**
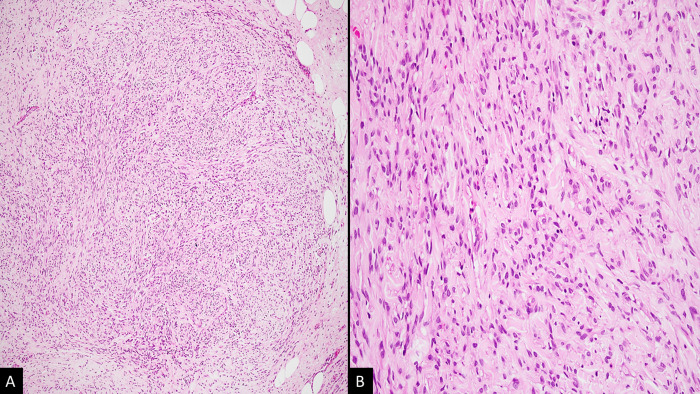
Histological images of the presented case. **A.** Mesenchymal neoplasm revealing vaguely nodular cellular regions admixed with variable amount of fat (H&E, 100X); **B.** cellular areas comprised of short, randomly intersecting fascicles of spindle cells, interspersed with keloid-like collagen fibres (H&E, 200X).

The gross examination of the specimen revealed a well-defined yellowish to grey-white lesion, measuring 6.2 x 5 cm. Importantly, all margins appeared unremarkable and devoid of any tumour involvement. The microscopic analysis of the lesion showed a benign mesenchymal neoplasm revealing vaguely nodular cellular regions admixed with variable amount of fat. The cellular areas comprised of short, randomly intersecting fascicles of spindle cells, interspersed with keloid-like collagen fibres. These spindle cells exhibited uniformity in size and displayed plump to elongated nuclei, with dispersed chromatin, small nucleoli, and scant pale to eosinophilic cytoplasm (Figure 1). A fair number of mast cells are also present. Mitotic figures, atypical mitoses, or necrosis were absent.

In view of the histomorphological characteristics, several differential diagnoses were considered, including solitary fibrous tumour, desmoid-type fibromatosis, low-grade myofibroblastic sarcoma, and low-grade fibromatosis-like metaplastic carcinoma. To establish a definitive diagnosis, we employed immunohistochemistry, utilizing a panel of markers that included AE1/AE3, androgen receptor (AR), CD34, desmin, estrogen receptor (ER), progesterone receptor (PR), p16, p63, and SMA.

**Figure 2:**
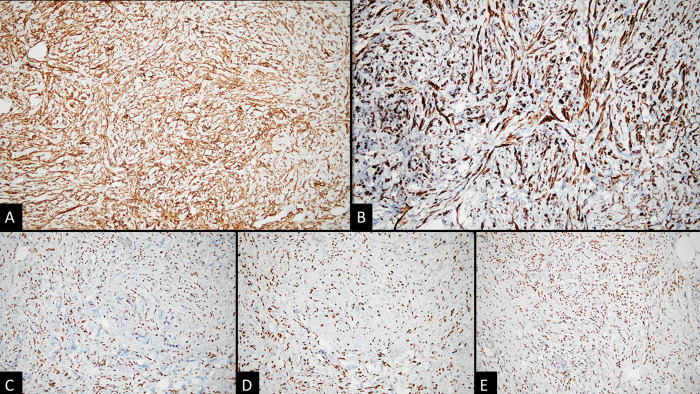
Immunohistochemical panel for the presented case. The neoplastic cells exhibit immunopositivity for A) CD34 (200X); B) Desmin (200X); C) ER (200X); D) PR (200X); E) AR (200X).

The immunohistochemical analysis revealed that the spindle cells exhibited positive staining for ER, PR, and AR, CD34, and desmin (Figure 2).

Conversely, they displayed immunonegativity for AE1/AE3, p63, p16 and smooth muscle actin (SMA). In accordance with the clinical presentation, radiological findings, histomorphological features, and immunohistochemical expression pattern, we arrived at a conclusive diagnosis of mammary myofibroblastoma. The patient is currently under observation and has been advised to follow up after three months.

## Discussion

Myofibroblastoma is a rare, benign mesenchymal breast tumour with a slight male predilection and is mainly documented in older men and postmenopausal women. It was initially identified in 1987 and over the years, there have been publications in the fields of pathology, surgery, and radiology about this uncommon neoplasm. Due to its infrequency, distinguishing between benign and malignant lesions in core biopsy specimens presents a significant challenge for pathologists, often requiring surgical excision for definitive diagnosis^1-3^.

Mammary myofibroblastoma typically manifests as a painless, slowly enlarging, and mobile mass. In the breast, these tumours seldom exceed 4 cm in size, with most measuring around 2 cm^1^. Initially, they were thought to be related to solitary fibrous tumour, but subsequent investigations revealed a closer association with spindle cell lipoma and vulvovaginal cellular angiofibroma. This distinction was based on the absence of STAT6 rearrangements and the presence of chromosomal deletions in the 13q14 region, which includes the RB1 and FOXO1A genes^1,4^. To gain a deeper understanding of myofibroblastoma's pathophysiology, it is essential to explore the molecular mechanisms governing myofibroblast growth and proliferation. Myofibroblasts respond to tissue injury, with cytokines like transforming growth factor beta-1 being produced by injured or potentially malignant cells. These cytokines facilitate the migration of fibroblasts into the injured tissue. Subsequently, smooth muscle actin fibres develop, ultimately transforming into myofibroblasts with contractile properties. This process contributes to the pathogenesis of myofibroblastoma^5^.

The imaging features of mammary myofibroblastoma lacks specificity. In mammography, these tumours usually appear as well-defined, lobulated, and hyperdense or isodense masses, without calcifications. MRI scans exhibit hyperintensity on T2-weighted images, isointensity on T1-weighted images, and exhibit heterogeneous enhancement^1,6^. Macroscopically, mammary myo-fibroblastoma usually appears as a well-circumscribed, round to oval mass. Its external surface is often smooth and may display lobulated contours. The tumour is characteristically firm in consistency. When the mass is sectioned, the cut surface typically reveals a solid lesion that appears pale white to greyish in colour. Additionally, the cut surface can take on a yellowish hue if there is an increased adipocytic component within the tumour. Notably, there are no signs of necrosis or haemorrhage within the tumour^1,3,7^. The microscopic examination of myofibroblastoma shows a well-defined, smoothly circumscribed border with the surrounding uninvolved breast tissue. Histologically, they are comprised of short spindle cells arranged in somewhat parallel fascicles. These cells are interspersed with brightly eosinophilic collagen, which can take on a broad and keloid-like appearance or appear thin and "ropey". The spindle cells within the lesion exhibit ovoid nuclei, pale eosinophilic cytoplasm with vague cytoplasmic borders, and inconspicuous vasculature. Mast cells are often present, and there may be occurrences of chondroid, osseous, or smooth muscle metaplasia. More importantly, there is no or minimal nuclear atypia observed. Mitotic figures are rare or absent, and there is no evidence of necrosis within the tumour. The adipocytic component within myofibroblastoma varies significantly and can be either absent or quite extensive^1,3,5^.

In terms of immunohistochemistry (IHC), myofibroblastoma typically exhibits a distinct expression profile. It shows positive staining for CD34, ER, PR, and AR. Additionally, consistent with its myofibroblastic lineage, it expresses desmin, SMA, and calponin^1,3^. It is worth noting that myofibroblastomas negative for CD34 are rare, and this includes the leiomyomatous variant^8^. As mentioned earlier in molecular terms, most cases of myofibroblastoma exhibit chromosomal deletions in the 13q14 region. Given that this region encompasses the Rb gene, the loss of Rb expression detected by immunohistochemistry can serve as a supportive adjunctive test in challenging cases^8^.

Amongst others, the most important mimicker of classic myofibroblastoma, particularly in core needle biopsies, is low-grade fibromatosis-like metaplastic carcinoma. Low-grade fibromatosis-like metaplastic carcinoma tends to be negative for CD34 and strongly positive for cytokeratin, especially high molecular weight cytokeratins, and p63. In contrast, myofibroblastoma consistently tests negative for these markers^1,3^.

The available data on myofibroblastoma from the Indian subcontinent is limited, primarily consisting of sporadic case reports^9-11^. Thus, this case report assumes significant importance as it supplements the current literature and addresses to the challenges faced in diagnosing mammary myofibroblastoma. This neoplasm can create a deceptive appearance of infiltrative growth at the interface between the spindle cells and intralesional adipocytes and even incorporate mammary glands; as was seen in our case. Consequently, it can mimic various infiltrative spindle cell neoplasms, including desmoid fibromatosis and dermatofibrosarcoma protuberans (DFSP). CD34 staining can be used to differentiate myofibroblastoma from desmoid fibromatosis, as it is almost always negative in the latter. On the other hand, nuclear expression of beta-catenin by immunohistochemistry supports the diagnosis of desmoid fibromatosis. DFSP, like myo-fibroblastoma, is diffusely positive for CD34 but reliably tests negative for ER and PR and does not exhibit Rb loss by IHC^1,3^.

Surgical excision is the primary treatment for mammary myofibroblastoma. The prognosis is generally favourable, with extremely low recurrence rates following complete removal. Long-term follow-up is recommended to monitor for potential recurrences^12^.

## Conclusion

Mammary myofibroblastoma, though rare, demands attention as a potential cause of breast lumps. Its elusive nature requires a multidisciplinary approach involving clinicians, radiologists, and pathologists to accurately diagnose and appropriately manage the condition. Familiarity with its mimics and differential diagnoses is essential to ensure optimal patient care. Given the scarcity of data from India, this case report contributes valuable insights to the understanding and recognition of this neoplasm.

## References

[1] Magro Gaetano (2016). Mammary myofibroblastoma: an update with emphasis on the most diagnostically challenging variants.. Histology and histopathology.

[2] Jung Hyun K, Son Jung H, Kim Woo G (2020). Myofibroblastoma of the breast in postmenopausal women: Two case reports with imaging findings and review of the literature.. Journal of clinical ultrasound : JCU.

[3] Magro Gaetano (2008). Mammary myofibroblastoma: a tumor with a wide morphologic spectrum.. Archives of pathology & laboratory medicine.

[4] Magro Gaetano, Righi Alberto, Casorzo Laura, Antonietta Torrisi, Salvatorelli Lucia, Kacerovská Denisa, Kazakov Dmitry, Michal Michal (2012). Mammary and vaginal myofibroblastomas are genetically related lesions: fluorescence in situ hybridization analysis shows deletion of 13q14 region. Human Pathology.

[5] Magro Gaetano (2009). Epithelioid-cell myofibroblastoma of the breast: expanding the morphologic spectrum.. The American journal of surgical pathology.

[6] Lee Eun Ji, Chang Yun-Woo, Jin Yoon Mi, Kim Nam Won (2018). Multimodality images of myofibroblastoma in the male breast: A case report and a review of the literature. Clinical Imaging.

[7] Howitt Brooke E., Fletcher Christopher D.M. (2016). Mammary-type Myofibroblastoma. American Journal of Surgical Pathology.

[8] D'Alfonso Timothy M., Subramaniyam Shivakumar, Ginter Paula S., Mosquera Juan Miguel, MacDonald Theresa Y., Noorzad Zohal, Orta Lurmag Y., Liu Yi-Fang, Rubin Mark A., Shin Sandra J. (2016). Characterization of the leiomyomatous variant of myofibroblastoma: a rare subset distinct from other smooth muscle tumors of the breast. Human Pathology.

[9] BHARATHI K (2014). Myofibroblastoma of Female Breast Masquerading as Schirrous Malignancy – A Rare Case Report with Review of Literature. JOURNAL OF CLINICAL AND DIAGNOSTIC RESEARCH.

[10] Khade ArchanaL, Khatib Yasmeen, Pandey Vinita, Pandey Rahul (2018). Myofibroblastoma of the Breast: A Rare Cause of Breast Lump in a Postmenopausal Woman. Journal of Mid-life Health.

[11] Talwar Amrita, Jain Swasti, Ahuja Arvind, Paliwal Purnima (2021). A Rare Case of Epithelioid Myofibroblastoma of Breast Mimicking Lobular Carcinoma on Trucut Biopsy: a Diagnostic Pitfall and Literature Review.. Indian journal of surgical oncology.

[12] Metry Mario, Shaaban Mohamad, Youssef Magdi, Carr Michael (2016). Myofibroblastoma of the Breast: Literature Review and Case Report.. Case reports in oncological medicine.

